# Floor-to-Stand Transfers in Older Adults: Insights into Strategies and Lower Extremity Demands

**DOI:** 10.3390/geriatrics10050119

**Published:** 2025-09-06

**Authors:** Lyndsay Stutzenberger, Tyler Whited

**Affiliations:** 1 Doctor of Physical Therapy Program, Division of Health and Behavioral Sciences, College of Allied Health, George Fox University, Newberg, OR 97312, USA; 2 Division of Health and Behavioral Sciences, Kinesiology Department, College of Allied Health, George Fox University, Newberg, OR 97312, USA; twhited@georgefox.edu

**Keywords:** floor-to-stand transfer, getting up from the floor, five times sit to stand, older adults

## Abstract

Background/Objectives: Getting up from the floor is an important functional skill for independence in older adults but is not often assessed clinically. The purpose of this study was to investigate how floor-to-stand transfer (FTS) ability is related to self-report measures and five-time sit-to-stand (5XSTS) performance, as well as compare peak joint angles during common FTS strategies and lower extremity demands between the 5XSTS and a commonly instructed FTS strategy. Methods: Thirty-four community-dwelling older adults completed self-report measures and performed the 5XSTS, a FTS in a self-selected manner, and an instructed FTS strategy. Biomechanical analysis of the lower extremities was used to determine peak joint angles, moments, and powers during study tasks. Correlation analyses were used to determine associations between FTS time and self-report scores, 5XSTS time, and lower extremity demands during the 5XSTS. One-way analysis of variance (ANOVA) and Kruskal–Wallis tests were used to determine the differences in self-report measures, 5XSTS performance, and FTS time between self-selected FTS strategies. Lower extremity demands between the 5XSTS and the instructed FTS strategy were compared with Wilcoxon Signed-Rank tests. Results: Self-report measures were not associated with FTS time or different between FTS strategies. Knee flexion was greater in the roll-over compared to the quadruped strategy (*p* < 0.001). Ankle and hip demands were greater during the instructed FTS, and knee demands were greater during the 5XSTS (*p* < 0.001) when comparing the tasks. Conclusions: The study findings may improve clinical decision-making related to FTS assessment and interventions in older adults. Prescribing exercises with greater hip and ankle demand than the 5XSTS may help maximize FTS ability.

## 1. Introduction

Maintaining independence at home is important as the US population continues to age and fall-related medical spending continues to increase [1,2,3,4]. Nearly half of older adults are unable to get up from the floor after a fall in the absence of an injury, and floor-to-stand transfer (FTS) ability is associated with falls, physical function, hospitalization, need for caregiver support, and even mortality [5,6,7,8,9,10]. Getting up from the floor, a task that becomes more difficult with age, is considered a geriatric functional milestone to routinely screen and identify functional impairments [7,11,12,13,14,15]. Unfortunately, the limited research available suggests clinicians infrequently assess the ability of their older patients to perform a floor-to-stand transfer [16,17].

Since FTS ability is associated with health status in older adults, FTS assessment and training is a vital component of patient care [5,6]. Self-report measures may assist clinicians with decision-making for safely and effectively integrating the FTS into clinical practice [6,9,18,19,20,21,22,23,24]. Previous research has shown that a patient’s self-reported FTS ability level (i.e., independent, assisted, or dependent) is associated with actual ability, and thus clinicians can use this information as a screening tool [6]. Clinicians may also consider using the Falls Efficacy Scale International (FES-I) questionnaire, common in clinical practice, since some research has shown older adults who had trouble getting up from the floor report greater fear of falling (FoF) [9,18,19,20,21,22,23,24]. Questionnaires from the Patient Reported Outcomes Measurement Information System (PROMIS) may also be helpful to understand other aspects of a patient that may relate to getting up from the floor, such as physical function and self-efficacy [25]. The measure of self-efficacy (SE) in managing daily activities, which is confidence in the ability to perform daily activities without assistance, has been shown to independently predict health outcomes [26,27]. Physical function (PF), which assesses a person’s view of their physical ability, has been validated across multiple patient populations and linked to fall risk [28,29]. Little is known, however, about how the FES-I and the PROMIS SE and PF self-report measures are associated with how quickly and in what way older adults get up from the floor. Since fear of falling and self-reported SE and PF may be associated with FTS ability, understanding these relationships could assist therapists in customizing FTS assessment and treatment.

In addition to self-reported FTS ability level, time to complete the five-time sit-to-stand test (5XSTS) can be used as a screening tool, since it has moderate sensitivity and strong specificity in predicting FTS ability level in older adults [13]. Thus, 5XSTS time is useful for making clinical decisions related to FTS assessment and interventions [6,13,30,31,32]. Except for time to complete the task, however, other factors related to performing the 5XSTS have not been studied in relation to FTS ability. Specifically, how biomechanical demands of the lower limbs are associated with FTS time and potentially differ in individuals who use different FTS strategies is unknown [6]. Such information would be clinically useful by providing insights into the role of the lower extremity joints in FTS ability, assisting clinicians in choosing exercises that best target impairments.

When older adults get up from the floor, three common strategies have been observed: getting up from a half-kneeling position, pushing up from a quadruped position to standing, and rolling forward and up to standing [14]. Despite the multiple strategies used by older adults, therapists most commonly teach the half-kneeling strategy [5,6,33,34,35]. To the authors’ knowledge, no FTS strategy has been found to be associated with greater or reduced risk of falling. However, instructing a single strategy, rather than one in which the patient is most comfortable or able to perform, could theoretically lead to difficulty in performing the task outside of the clinical setting or even avoiding it. Information that would help therapists anticipate the strategy a patient might use could improve patient safety, success, and confidence in the task with tailored interventions. Potential tools to help clinicians anticipate FTS strategies include self-report questionnaires (i.e., FES-I, SE, and PF) that may differ in individuals who choose different FTS strategies. Common strategies may also involve different biomechanical demands that could be clinically useful to understand. Lastly, any differences in biomechanical demand of the lower limbs between the commonly instructed half-kneeling FTS strategy and a sit-to-stand activity (i.e., 5XSTS test) could assist clinicians in prescribing exercises to improve FTS ability.

This study had multiple aims. The first aim was to study the relationships between FTS time and age, 5XSTS time, scores on self-report measures, and lower limb demand during the 5XSTS. Longer self-selected FTS times were hypothesized to be associated with greater age, longer 5XSTS time, higher FES-I scores, lower SE and PF scores, and decreased lower limb demand during the 5XSTS task. The second aim was to study the differences in participants who performed different FTS strategies. Specifically, self-report questionnaire scores, time to complete the FTS and 5XSTS tasks, lower limb demand during the 5XSTS, and joint angles during the self-selected FTS were compared. It was hypothesized that differences would be found when comparing different self-selected FTS strategies. The last aim was to study potential differences in lower limb biomechanical demands between the 5XSTS and instructed FTS with the hypothesis that demands would be greater in the FTS.

## 2. Materials and Methods

### 2.1. Participant Selection and Consent

A convenience sample of participants was recruited from November 2023 to May 2024 in the surrounding area through fliers and word of mouth. To participate, individuals needed to be 50–80 years old, living independently in the community or an assisted living facility, able to walk at least 15 feet, speak English, and self-report willingness to attempt to get up from a chair and the floor. Participants were excluded if the Saint Louis University Mental Status Examination (SLUMS) indicated dementia, with a cut-off score of 21 or lower for those with at least a high school education or a score of 20 or lower for those with less than a high school education [36]. Participants were also excluded if they self-reported a neurologic condition, unstable angina, or joint replacement or major surgery in the previous six months. Additional exclusion criteria included reporting a concussion in the last 3 months, dizziness in the past 2 weeks, current severe pain rated 7/10 or greater, or any condition that would prevent participants from getting up and down from the floor safely. Lastly, resting vital signs needed to be within limits considered safe for physical activity [37,38].

An a priori analysis was conducted (G*Power, Version 3.1.9.6, Kiel, Germany) to determine the sample size needed for this study. Using a one-way ANOVA with fixed effects to determine an effect size of 0.4 at an alpha level of 0.05 and a power of 0.8, 30 participants were found necessary to determine differences between the three commonly performed FTS strategies used by older adults.

### 2.2. Study Procedures

#### 2.2.1. Self-Report Measures

Participants filled out self-report questionnaires that included the Falls Efficacy Scale International (FES-I), the Rapid Assessment of Physical Activity (RAPA), and Patient Reported Outcome Measurement Information System (PROMIS) measures of self-efficacy during daily activities (SE) and physical function (PF). The FES-I was chosen because it is commonly used clinically to measure FoF in older adults, and FoF is associated with difficulty getting up from the floor after a fall [9,18,19,20,21,22,23,24]. The FES-I is a valid and reliable measure with 16 questions scored on a 4-point scale from 1 (not at all concerned) to 4 (very concerned) that total to indicate low (16–19), moderate (20–27), and high (28–64) levels of concern for falling [18,19,20,21,23]. The RAPA, a valid and reliable measure of physical activity levels in older adults, was completed to describe participant activity levels [39,40]. Items from the RAPA 1 section of the questionnaire measure aerobic activity, and RAPA 2 items measure strength and flexibility activity [39,40]. The PROMIS measures of SE and PF were chosen because they are patient-centered measures that provide information related to patient perception of abilities [41,42,43]. These PROMIS measures are computer adaptive tests (CAT), which use a bank of questions that are individualized and produce a T-score [42,43]. A T-score of 50 equates to the mean of the general U.S. population, except for SE, which is normed to a clinical population with chronic conditions [41,42]. The measure of PROMIS SE assesses a person’s confidence in their ability to perform various daily activities without assistance [42]. The measure of PROMIS PF assesses self-reported physical capability [43]. Both measures of PROMIS SE and PF have been validated across diverse patient populations [26,28,44,45,46].

#### 2.2.2. Study Activities

Participant height and weight were measured and recorded. Participants wore a gait belt and performed the 5XSTS with a 5 minute break before completing a FTS task in a self-selected manner (self-selected FTS). If participants performed the self-selected FTS task independently, they also performed a FTS task in a commonly instructed manner (instructed FTS). Participants verbally reported their rate of perceived exertion (RPE) after the 5XSTS and self-selected FTS tasks. All study movements were recorded with 11 cameras (Qualysis, Goteborg, Sweden) at 120 Hz. Two force plates (AMTi, Watertown, MA, USA) recording at 1200 Hz captured force data during the 5XSTS and instructed FTS tasks.

#### 2.2.3. 5XSTS Task

Participants performed a 5XSTS task, previously shown to predict FTS ability level, as described in previous research and typically performed by older adults in clinical settings [13,30]. Participants stood up and sat down as fast as possible from a standard height chair (18′′) with arms crossed over their chest while guarded by a researcher [13]. Time was recorded from the researcher’s verbal command “go” until the participant touched the chair after the 5th repetition [30].

#### 2.2.4. Self-Selected FTS Task

**Figure 1:**
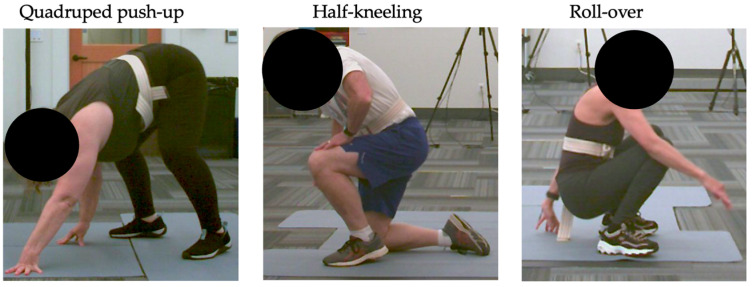
Floor-to-stand transfer strategies performed by study participants based on the position observed while transitioning to standing, which included the quadruped push-up (**Left**), half-kneeling (**Center**) and roll-over (**Right**) strategies.

Since older adults get up from the floor in a variety of ways, and FTS ability is associated with health status, participants were asked to perform a timed FTS using a self-selected FTS strategy with a researcher standing nearby [5,6,14,33,34,35]. Participants were instructed to lie on the floor on their back. On the researcher’s verbal command of “go”, participants were instructed to stand up as quickly as possible in any way they chose [5,14]. Time was recorded from the researcher’s verbal start until the participant achieved their full balanced standing height. A second researcher characterized the transfer by level of independence (i.e., independent, assisted, or dependent) and strategy used. The FTS strategy was identified based on the position observed while moving to standing, regardless of the movement patterns observed prior to standing up. Strategies were characterized as half-kneeling (half-kneel), roll-over, or quadruped push-up (quadruped), per published definitions [5,14] (Figure 1). The primary investigator characterized strategies initially and consulted a second researcher when transfer strategies were not immediately apparent.

#### 2.2.5. Instructed FTS Task

Participants watched a video demonstrating and describing a standardized FTS using the half-kneeling strategy commonly performed by older adults and instructed by clinicians during FTS training [5,6,33]. Participants were verbally cued to position the lead foot on a single force plate to record forces through that limb. Participants were also instructed to not use their hands when moving from the half-kneeling position to standing. Lead limb position and absence of upper limb support when standing up were necessary to quantify lower limb demand through biomechanical analyses. Participants repeated this task until three trials were successfully recorded as instructed, 5 total trials were performed, or the participant ended their participation. Rest between trials was given as needed.

### 2.3. Data Analysis

Markerless motion capture videos were processed using Theia3D (version 2023, patch 18, Theia Markerless, Kingston, ON, Canada) to create a biomechanical model of the upper and lower limbs, pelvis, and trunk. Theia3D uses a deep-learning algorithm to locate anatomical landmarks without the need for reflective markers to be placed on the body, as required with traditional marker-based motion capture systems [47,48]. Using a markerless motion capture system was a practical and necessary choice for recording a task such as the FTS transfer [49,50]. Reflective markers necessary on the back of the body to create a biomechanical model from a marker-based system would be hidden during the initial part of the task, uncomfortable to lie directly on, and unlikely to remain on the body throughout the task. Theia3D has been shown to be reliable and concurrently valid with traditional marker-based systems for analyzing lower body movements in the sagittal plane during tasks such as running, walking, jumping, and squatting [49,50,51,52,53,54].

Further analysis was performed using Visual3D (version 2024.6, HAS-Motion Inc., Kingston, ON, Canada). Kinematic and force data were filtered with a cut-off frequency of 6 Hz. Joint angles of the hip, knee, and ankle were calculated, defined as the distal segment relative to the proximal segment. Inverse dynamics was performed to calculate ankle, knee, and hip joint torques and powers from position, anthropometric, and force plate data.

During the 5XSTS and self-selected FTS tasks, peak dorsiflexion, knee flexion, and hip flexion angles were identified from either limb. Peak joint angles were also identified from the lead limb during the instructed FTS task. Peak internal joint torques and power, normalized to body mass, for ankle plantar flexion, knee extension, and hip extension were identified from either limb during the 5XSTS and the lead limb during instructed FTS task. Peak values of biomechanical variables were averaged across the three successful trials of the instructed FTS task.

Biomechanical data was not available for two participants for the 5XSTS task due to technical difficulties, with one of these participants also missing biomechanical data from the instructed FTS due to inability to perform the task without upper limb support. Biomechanical data on the instructed FTS was not available from eight additional participants due to factors occurring in isolation or combination that included inability to perform all trials without using the upper limbs, foot placement that was not isolated on a force plate, and voluntarily ending the study session before completion of all activities. Thus, biomechanical data from these participants were not included in the relevant statistical analyses.

### 2.4. Statistical Analysis

Normality testing was performed on scores from self-report measures and biomechanical variables during the study tasks using skewness and kurtosis values, with >3 indicating a violation of normality assumptions. Some biomechanical variables related to the 5XSTS and instructed FT task kinetics violated normality assumptions and required nonparametric testing for analyses. To determine any association between self-selected FTS time and age, as well as 5XSTS time and scores on self-report measures, Pearson correlations were performed. Spearman correlations were performed to identify any association between self-selected FTS time and 5XSTS lower extremity joint torques and powers. To determine differences in older adults who chose the various FTS strategies, self-report measure scores, 5XSTS time, self-selected FTS time, and peak lower extremity joint angles during the self-selected FT were each compared using one-way analysis of variance (ANOVA). When the omnibus ANOVA was significant (*p* < 0.05), independent *t*-tests were performed between pairs of the three FTS strategies. To determine differences in lower extremity joint torques and powers during the 5XSTS task between pairs of self-selected FTS strategies, Kruskal–Wallis tests were performed. When the omnibus Kruskal–Wallis tests were significant (*p* < 0.05), post-hoc Dunn’s testing was performed. Lastly, to compare lower extremity demands between the 5XSTS and instructed FTS tasks, Wilcoxon Signed-Ranks tests were used to determine differences in lower extremity peak joint angles, moments, and powers. Statistical tests were Bonferroni corrected to account for the multiple tests performed. Statistical tests were performed using SPSS (Version 27; IBM Corp., Armonk, NY, USA).

## 3. Results

### 3.1. Participants and Study Activity Performance

Thirty-five community-dwelling older adults provided informed consent to participate in study activities, of which 34 (13 men and 21 women) with a mean age of 69 (SD = 9) years met the study’s eligibility criteria. Participants self-reported their race/ethnicity as 91.2% White, 5.9% Hispanic, and 2.9% Asian. Participant characteristics are reported in Table 1. Mean scores on the FES-I indicated a low level of FoF on average, with 79% and 21% of participants categorized as having low (score of 16–19 out of 64) and moderate (score of 20–27 out of 64) concern for falling, respectively [18,19,20,21,23]. Mean RAPA 1 scores were 6.2 (SD = 0.8), with 85% of participants categorized as “active,” indicated by a score of 6–7 out of 7 points [39,40]. Mean RAPA 2 scores out of a possible 3 points were 2.2 (SD = 1.1), with 88% of participants performing strength and/or flexibility exercises weekly, indicated by a score of at least 2 points. Mean (SD) PROMIS scores, referenced to a mean 50-point T-score, were 54.2 (4.9) for SE and 53.7 (6.5) for PF, indicating slightly higher scores than the reference population average [41]. Mean (SD) 5XSTS and self-selected FTS times were 9.6 (1.7) and 4.7 (2.1) seconds, respectively (Table 2). All participants performed the self-selected FTS task independently, with 17 (50%), 11 (32.4%), and 6 (17.6%) using the half-kneel, quadruped, and roll-over strategies, respectively.

### 3.2. Self-Selected FTS Time Correlations

As depicted in Table 2, longer self-selected FTS times were significantly and moderately associated with greater age (r = 0.52, *p* = 0.002) and longer 5XSTS times (r = 0.56, *p* < 0.001). No associations were found between self-selected FTS time and the self-report measures used in this study.

### 3.3. Differences Between Participants Who Performed Various FTS Strategies

#### 3.3.1. Self-Report Measures, Self-Selected FTS Time, and 5XSTS Performance

No significant differences were found in self-report measures, FTS time, 5XSTS time, or lower extremity joint torques and powers during the 5XSTS task between participants who used different self-selected FTS strategies (Table 3).

#### 3.3.2. Joint Angles During Self-Selected FTS

**Figure 2:**
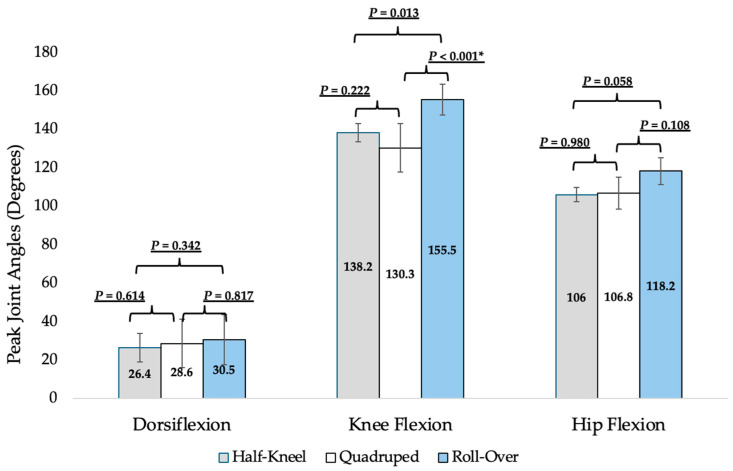
Peak joint angles with mean (SD) and *p*-values for differences found in independent *t*-tests between participants who used different floor-to-stand transfer strategies. * Indicates significance level below Bonferroni-corrected *p*-values for post-hoc testing of differences between strategies.

Significant differences in peak joint angles of the lower extremities were found during performance of the self-selected FTS strategies (Figure 2). Peak knee flexion angles during the roll-over strategy were significantly greater than the quadruped strategy by an average of 25.2 degrees (*p* < 0.001).

### 3.4. Lower Extremity Demands Between 5XSTS and Instructed FTS

**Figure 3:**
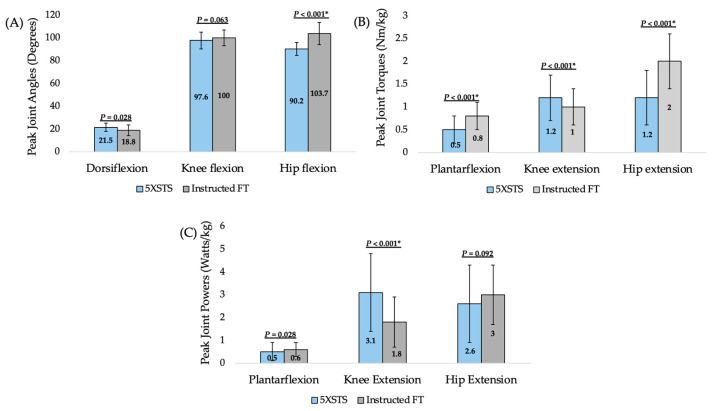
(**A**) Peak joint angles with mean (SD) during the 5XSTS and instructed FTS with *p*-values for differences found in Wilcoxon Signed-Rank tests between the tasks; (**B**) Peak joint torques with mean (SD) during the 5XSTS and instructed FTS with *p*-values for differences found in Wilcoxon Signed-Rank tests between the tasks; and (**C**) Peak joint powers with mean (SD) during the 5XSTS and instructed FTS with *p*-values for differences found in Wilcoxon Signed-Rank tests between the tasks. Abbreviations: 5XSTS = five-time sit-to-stand; instructed FTS = instructed floor-to-stand; Nm = Newton meter; kg = kilogram. * Indicates significance level below Bonferroni-corrected *p*-values for post-hoc testing of differences between strategies.

Significant differences were found in lower extremity peak joint angles, torques, and powers between the 5XSTS and instructed FTS tasks in the 24 participants with biomechanical data available for both tasks (Figure 3). Peak hip flexion was significantly greater in the instructed FTS task than the 5XSTS task (*p* < 0.001). Peak ankle plantar flexion torque (*p* < 0.001), peak knee extension torque and power (*p* < 0.001), and peak hip extension torque (*p* < 0.001) also differed significantly between the tasks. Ankle and hip demands were generally greater in the instructed FTS, while knee demands were generally greater in the 5XSTS task.

## 4. Discussion

Getting up from the floor is a key functional movement for independence in older adults and an important part of screening for declining mobility [6,7,11,12,14,15]. Unfortunately, assessment of the often-difficult task of getting up from the floor in older adults is not frequently performed, limiting opportunities to address related impairments for maximizing patient safety and quality of life [16,17]. Evidence-based guidance for anticipating how quickly and in what way a patient might get up from the floor, along with an understanding of differences in lower extremity demand between the 5XSTS and the half-kneeling FTS strategy, may improve FTS integration into clinical practice. Based on the study findings, neither self-selected FTS speed nor strategy can be anticipated with commonly used self-report measures or the 5XSTS test. Differences found in lower limb demands between the 5XSTS and the half-kneeling FTS strategy (Figure 3) and in knee mobility between FTS strategies (Figure 2) can assist with FTS training and interventions. Overall, the study findings provide evidence for clinical decision-making related to FTS assessment, instruction, and exercise prescription.

Study findings showed a significant and moderate association of FTS time with age and 5XSTS time. Previous studies have also shown an association between longer FTS times and greater age and decreased lower limb strength as indicated by longer 5XSTS times [6,7,12,15]. Time to complete the self-selected FTS transfer in this study ranged from 2 to 10.7 s, with an average of 4.7 (SD 2.1) seconds, consistent with that previously demonstrated in healthy older adults [12,14]. All participants in this study performed the 5XSTS in under 13.6 s, a cut-off time previously shown to predict the ability to perform a FTS without using a chair [5,6]. Overall, this study’s findings support previous research and further highlight the associations between both 5XSTS time and age to FTS ability.

No self-report measures utilized in this study were significantly associated with self-selected FTS time, though previous research has shown FoF to be associated with difficulty getting up from the floor [9,18,19,20,21,22,23,24]. This study included an active population with self-reported SE and PF above established norms, low FoF, and physical performance tests within age-related norms. Thus, in a healthy older adult population with relatively high SE and baseline physical activity, FTS time may be discriminated more by physical ability than patient perceptions of self. Emphasis in clinical practice should remain focused on building and preserving physical capacity to perform the FTS task in active older adults [5,6,7,8,9,15,22,43,44,45,46]. This study also did not collect information on participants’ fall history, which may have been a factor in this study’s findings. Further research is warranted to investigate relationships between FTS abilities and self-report measures in older adults with more broad ranges (i.e., spanning lower and higher scores) for SE, PF, and FoF responses and 5XSTS performance.

Based on this study’s findings, various self-selected FTS strategies involve different peak knee joint angles. Participants who performed the quadruped strategy demonstrated significantly less knee flexion than those who performed the roll-over strategy. Results suggest that clinicians may be able to anticipate the use of the quadruped FTS strategy in community-dwelling older adults with less knee mobility. Clinical judgment is necessary to make decisions related to tailoring interventions to a self-selected FTS strategy or training the more commonly instructed half-kneel strategy. Further research is warranted to understand why certain strategies are preferred and study other factors, such as balance and lower limb strength and power, that may differ in individuals who self-select different FTS strategies.

This study’s findings indicate that the 5XSTS and instructed FTS tasks are biomechanically different when upper limb use is eliminated in both tasks. Greater demand at the ankle and hip was found in the instructed FTS, and greater demand at the knee was found during the 5XSTS. The instructed FTS involved greater peak hip flexion angles and hip extension and ankle plantarflexion torque than the 5XSTS. The 5XSTS utilized greater peak knee extension torque and power than the instructed FTS task. Research supports the use of the 5XSTS to reliably predict FTS ability; however, the lower extremity demands of the 5XSTS do not entirely mimic that of the commonly instructed half-kneeling FTS strategy when performed without upper limb assistance. Though some older adults may not be able to get up from the floor without upper limb support, which was observed in this study, circumstances may arise that require FTS without using the upper limbs (e.g., picking up an object with both arms and upper limb injury). Thus, it is imperative to understand the lower limb demands during an FTS performed without using the upper limbs. Based on this study’s findings, clinicians may consider prescribing exercises that increase the hip and ankle strength and power demands beyond that of the 5XSTS to maximize FTS ability. Further research is warranted to investigate the role and contributions of the upper limbs when used to get up from the floor.

This study is not without limitations. Participants were from a convenience sample of community-dwelling and active older adults 50–80 years old without significant cognitive impairment. All participants performed the self-selected FTS task independently. Thus, this study’s findings cannot be applied to other populations of different age ranges, physical activity levels, health status, or FTS ability levels. This study defined three commonly used FTS strategies in older adults based on the body position prior to standing. Strategy classification may therefore differ from studies that define the FTS strategy based on movements performed earlier in the FTS transfer. This study also compared the lower limb demands between the 5XSTS test and a single, commonly used and instructed FTS strategy, preventing comparisons to other FTS strategies performed by older adults. Lastly, this study did not allow upper limb assistance when standing up from the half-kneeling position during the instructed FTS task to isolate and quantify lower limb demand through biomechanical analysis. Further research is warranted to investigate the effects of upper limb support on FTS performance.

## 5. Conclusions

This study’s findings suggest differences in peak knee joint flexion between FTS strategies in older adults, with the greatest knee flexion in the roll-over strategy. Additional differences found in lower extremity biomechanical demands between the 5XSTS and the half-kneeling FTS strategy provide direction for optimizing exercise prescription to meet the physical demands of getting up from the floor. Exercises that challenge the hips and ankles more than during the 5XSTS task are recommended to improve the ability to perform the half-kneel FTS strategy. Overall, this study’s results provide evidence-based guidance for FTS training and exercise prescription to maximize functional independence in older adults.

## Figures and Tables

**Table 1:** Participant characteristics (N = 34).

Characteristics	Mean (SD)
Age (years)	69.1 (8.8)
Mass (kg)	74.4 (15.3)
Height (cm)	168.4 (11.4)
BMI (kg/m^2^)	25.7 (4.22)
SLUMS Score	27.1 (0.1)
FES-I Score	18.1 (2.0)
PROMIS SE T-score	54.2 (4.9)
PROMIS PF T-score	53.7 (6.5)
RAPA 1 score: Aerobic	6.2 (0.8)
RAPA 2 score: Strength and Flexibility	2.2 (1.1)
5XSTS time (seconds)	9.6 (1.7)
Self-selected FTS time (seconds)	4.7 (2.1)

Abbreviations: kg = kilograms, cm = centimeters, SLUMS = Saint Louis University Mental Status Examination, FES-I = Falls Efficacy Scale International, PROMIS = Patient Reported Outcome Measurement Information System, SE = self-efficacy, PF = physical function, RAPA = Rapid Assessment of Physical Activity, 5XSTS = five-time sit-to-stand, FTS = floor-to-stand.

**Table 2:** Correlations of age, self-report measure scores, and 5XSTS performance measures to self-selected FTS time with *p*-values found in Pearson and Spearman correlation testing.

Self-Report and 5XSTS Performance Measures	Correlation to FT Time
Age (years)	r = 0.52, *p* = 0.002 *
PROMIS SE (T-score)	r = −0.27, *p* = 0.125
PROMIS PF (T-score)	r = −0.33, *p* = 0.060
FES-I Score	r = 0.19, *p* = 0.271
5XSTS time (seconds)	r = 0.56, *p* < 0.001 *
5XSTS ankle plantar flexion torque (Nm/kg)	r = −0.08, *p* = 0.676
5XSTS ankle plantar flexion power (W/kg)	r = −0.30, *p* = 0.098
5XSTS knee extension torque (Nm/kg)	r = −0.27, *p* = 0.139
5XSTS knee extension power (W/kg)	r = −0.46, *p* = 0.009
5XSTS hip extension torque (Nm/kg)	r = −0.30, *p* = 0.095
5XSTS hip extension power (W/kg)	r = −0.35, *p* = 0.050

Abbreviations: PROMIS = Patient Reported Outcome Measures Information System, SE = Self-Efficacy, PF = Physical Function, FES-I = Falls Efficacy Scale International, 5XSTS = five-time sit-to-stand, FTS = floor-to-stand. * Indicates significance level below Bonferroni-corrected *p*-values.

**Table 3:** Self-report and physical performance measures with mean (SD) of participants who used different self-selected FTS strategies.

	Half-Kneel	Quadruped	Roll-Over
Self-Report Measures			
PROMIS Self-Efficacy in Daily Activities (T-score)	56.02 (5.40)	51.84 (3.90)	53.20 (2.82)
PROMIS Physical Function (T-score)	53.62 (7.32)	51.38 (5.37)	57.98 (4.29)
FES-I score	17.47 (1.33)	19.36 (2.50)	17.33 (1.37)
Physical Measures			
FTS time (seconds)	4.04 (1.35)	6.26 (2.13)	3.68 (2.70)
5XSTS time (seconds)	9.29 (1.48)	10.36 (1.44)	9.36 (2.46)
5XSTS peak ankle plantar flexion torque (Nm/kg)	0.52 (0.32)	0.49 (0.18)	0.49 (0.30)
5XSTS peak ankle plantar flexion power (W/kg)	0.50 (0.31)	0.45 (0.29)	0.74 (0.67)
5XSTS peak knee extension torque (Nm/kg)	1.31 (0.52)	1.10 (0.33)	1.26 (0.55)
5XSTS peak knee extension power (W/kg)	3.47 (2.04)	2.42 (0.81)	3.12 (1.24)
5XSTS peak hip extension torque (Nm/kg)	1.17 (0.66)	1.13 (0.52)	1.20 (0.64)
5XSTS peak hip extension power (W/kg)	2.61 (1.69)	2.38 (1.50)	2.72 (1.63)

Abbreviations: PROMIS = Patient Reported Outcome Measures Information System, FES-I = Falls Efficacy Scale International, FTS = floor-to-stand, 5XSTS = five-time sit-to-stand.

## Data Availability

The raw data supporting the conclusions of this article will be made available by the authors on request.

## Abbreviations

The following abbreviations are used in this manuscript:

FT	Floor transfer
5XSTS	5-time sit-to-stand
ANOVA	One-way analysis of variance
FES-I	Falls Efficacy Scale International
FoF	Fear of Falling
SE	Self-efficacy
PF	Physical function
SLUMS	Saint Louis University Mental Status Exam
PROMIS	Patient Reported Outcome Measurement Information System
RAPA	Rapid Assessment of Physical Activity
CAT	Computer Adaptive Testing
